# Bridging the gap: a call for a joint paediatric-adult respiratory transition pathway

**DOI:** 10.3389/fmed.2026.1964085

**Published:** 2026-09-07

**Authors:** Sara Manti, Paolo Ruggeri

**Affiliations:** 1 Pediatric Unit, Department of Human Pathology in Adult and Developmental Age “Gaetano Barresi”, University of Messina, Messina, Italy; 2 Pulmonology Unit, Department of BIOMORF, University of Messina, Messina, Italy

**Keywords:** adult pulmonology, chronic respiratory disease, integrated care pathway, paediatric pulmonology, transition of care

## 1 Introduction

Advances in diagnosing, management, and treatment have significantly extended the lifespan of numerous children suffering from chronic respiratory conditions such as cystic fibrosis, severe asthma, bronchopulmonary dysplasia, non-cystic fibrosis bronchiectasis, neuromuscular disorders, and interstitial lung disease (1). However, this achievement introduces a new clinical challenge: ensuring a safe and effective transition for these patients from pediatric to adult respiratory care. The transition period, which has not yet been universally defined, although it is generally considered to coincide with adolescence, is increasingly recognized as a time of clinical vulnerability, rather than a mere administrative handoff (2). If managed inadequately, it is associated with loss of follow-up, reduced adherence to treatment, and long-term outcomes that can affect health outcomes in adulthood (3).

Despite growing awareness, transition care in respiratory medicine remains largely informal and clinician-dependent rather than structured and institutionalised. A survey of United States paediatric pulmonology programmes managing respiratory-technology-dependent patients found that the large majority did not use a standardised transition process, and fewer than a quarter tracked patients through to their first adult appointment (4). General evidence-based guidance on paediatric-to-adult transition across chronic conditions similarly notes that formal protocols remain the exception rather than the rule (5). Disease-specific data reinforce this picture. Preliminary data reported by the Child-BEAR-Net ERS Clinical Research Collaboration, currently available only as a conference abstract, suggest that patients with non-cystic fibrosis bronchiectasis receive less structured transition support than those with cystic fibrosis, with fewer scheduled pre- and post-transition visits and higher rates of missing transition documentation, despite comparable or worse lung function at the time of transfer; confirmation in peer-reviewed studies is still needed (6). This reflects how the quality of transition is currently shaped more by disease-specific investment and historical infrastructure than by patient need.

This significant gap was recently highlighted in the ERS statement on the transition pathway for pediatric patients with interstitial lung diseases, in which validated transition models from cystic fibrosis and diabetes care are adapted. It is precisely from this observation that we must begin anew, moving away from *ad hoc* solutions to finally embrace a structured and pragmatic approach (7).

Far from being a minor administrative nuance, this variability creates a profound fracture in the continuity of care. It occurs at a critical developmental juncture, precisely as adolescents are expected to assume autonomy over their complex therapeutic regimens, a shift that significantly elevates both the clinical and psychosocial stakes.

When transitional pathways rely solely on a referral letter, a single joint consultation, or informal peer-to-peer exchanges, the entire process becomes vulnerable to the goodwill and capacity of individual clinicians 1, rather than being anchored in standardized, institutionalized protocols. Consequently, care continuity becomes fragmented, leaving patients vulnerable to disengagement, loss to follow-up, and adverse long-term health outcomes during critical care transitions. In our view, such an approach is inherently non-standardized, unverifiable, and inequitable; consequently, clinical outcomes depend less on the severity of the disease and more on whether or not a patient is cared for by a well-integrated clinical team. This lack of formalization precludes the collection of reproducible process and outcome data, leaving clinicians unable to demonstrate tangible value to healthcare administrators and payers. Consequently, this evidentiary gap perpetuates the systemic underinvestment that currently cripples transition services (3). What is needed is a shift from transition as an individual clinical courtesy to transition as a formalised, institutionally embedded Paediatric-Adult Care Pathway in transition care in respiratory medicine.

Accordingly, a robust, integrated respiratory care pathway must be built upon a formalized architecture. Rather than relying on rigid chronological cut-offs, the entry into this pathway should be governed by clear timing and readiness criteria, dynamically triggered by clinical stability and the patient's phycological maturity. Operationally, this requires a structured phases characterized by identification and preparation, active transition, consolidation, and follow-up post transition. During this route paediatric and adult pneumology teams collaborate in a structure and close manner. Such frameworks facilitate the implementation of versatile, cross-cutting approaches tailored to the specific demands of any respiratory disease undergoing clinical transition (7–9). Clinical continuity demands a standardized data handover. This is best achieved through a shared summary template detailing the diagnosis, specific phenotype, therapeutic history, longitudinal lung function trajectories, and psychosocial variables. Our model, emphasizes the importance of monitoring lung function throughout this process from the outset, to identify the different functional trajectories a patient may follow during adolescence by detecting, at an early stage, functional biomarkers that can predict future outcomes in adulthood, thereby enabling therapeutic decisions, which are often made too late, to be planned. Beyond documentation, organizational clarity requires an unequivocal designation of responsibilities among paediatric and adult pulmonologists, ideally supported by a dedicated transition coordinator or case manager tasked with navigating the patient through this vulnerable gap. While ideally universal, this pathway must initially target priority disease groups with a documented high risk of being lost to follow-up; these include cystic fibrosis, severe or difficult-to-treat asthma, bronchopulmonary dysplasia, non-cystic fibrosis bronchiectasis, neuromuscular diseases with respiratory involvement, and childhood interstitial lung disease. Finally, to transition from an ad-hoc arrangement to an auditable medical standard, the pathway must embed objective process and outcome indicators. Tracking metrics such as attendance rates at the initial adult appointment, time to loss of follow-up, treatment adherence, exacerbation frequencies, and post-transfer lung function trends will provide the empirical data necessary for continuous quality improvement and institutional justification.

## 2 Discussion

We believe the evidence is now sufficient to move beyond further descriptive studies documenting the transition gap, and to act.

We call on:

1. Joint Professional Guidance: paediatric and adult respiratory and allergy societies must collaborate to issue practical guidelines for the design and implementation of respiratory transition pathways, effectively building upon existing disease-specific statements.
2. Institutional Recognition and Resourcing: hospital and regional health authorities need to formally recognize and resource joint transition clinics as a standardized framework of care, moving away from a discretionary service that relies entirely on the goodwill of individual clinical champions.
3. Core Curriculum Integration: training programmes in both paediatric and adult pulmonology should explicitly incorporate transitional medicine into their core specialist curricula, ensuring that structured care coordination becomes a mandatory competency expected of every graduating specialist.
4. Research networks: research networks must prioritize the multicentre evaluation of respiratory transition pathways implementation using shared process and outcome indicators, thereby demonstrating the clinical and economic value of structured transition with the same methodological rigour applied to other major quality-of-care interventions.

In conclusion, a formalized joint transition pathway is not an administrative luxury, but a crucial structural prerequisite for ensuring equitable, continuous, and accountable care. For young people navigating chronic respiratory diseases, this framework transforms the transition into adult medicine from a hazardous cliff edge into a secure, well-engineered bridge.

## 3 Key points

- Critical transition from paediatric to adult respiratory care is still frequently managed through informal, clinician-dependent arrangements that lack standardization.
- Transitional care in respiratory medicine must evolve from a series of fragmented handovers into a formalized, institutionally embedded framework of joint paediatric-adult integrated care.
- A call to action targeted at scientific societies, healthcare authorities, and academic training programmes.
- The proposed pathway builds on, and makes explicit its respiratory-specific additions to, established general transition-of-care frameworks.
- Respiratory scientific societies (both paediatric and adult), hospital administrators, policymakers and medical residency schools should move from informal transition practices toward foundation of the structural pillars of an effective transition pathway.
